# Author Correction: Molecular rearrangement of bicyclic peroxy radicals is a key route to aerosol from aromatics

**DOI:** 10.1038/s41467-023-44213-y

**Published:** 2023-12-14

**Authors:** Siddharth Iyer, Avinash Kumar, Anni Savolainen, Shawon Barua, Christopher Daub, Lukas Pichelstorfer, Pontus Roldin, Olga Garmash, Prasenjit Seal, Theo Kurtén, Matti Rissanen

**Affiliations:** 1 Aerosol Physics Laboratory, Tampere University, FI-33101 Tampere, Finland; 2 Department of Chemistry, University of Helsinki, P.O. Box 55, FI-00014 Helsinki, Finland; 3 Pi-Numerics, 5202 Neumarkt am Wallersee, Austria; 4 Department of Physics, Lund University, P.O. Box 118, SE-221 00 Lund, Sweden; 5 Swedish Environmental Research Institute IVL, SE-211 19 Malmö, Sweden; 6 Department of Atmospheric Sciences, University of Washington, Seattle, WA USA

**Keywords:** Reaction kinetics and dynamics, Atmospheric chemistry, Atmospheric chemistry

Correction to: *Nature Communications* 10.1038/s41467-023-40675-2, published online 17 August 2023

In the original version of this Article the chemical structures of P-C_1_ and P-C_2_ in Figure 1a were mistakenly inverted. This has been corrected in both the PDF and HTML versions of the Article.

