# Supplementary material for: Molecular rearrangement of bicyclic peroxy radicals is a key route to aerosol from aromatics
Source: Nat Commun. 2023 Aug 17;14:4984. doi: 10.1038/s41467-023-40675-2 (PMC10435581; doi:10.1038/s41467-023-40675-2)
Supplement: Supplementary file 1 — Supplementary Information [file 41467_2023_40675_MOESM1_ESM.docx]

**Supplementary** **Information to:**

**Molecular rearrangement of bicyclic peroxy radicals is a key route to aerosol from aromatics**

Siddharth Iyer^1∗^, Avinash Kumar^1^, Anni Savolainen^1^, Shawon Barua^1^, Christopher Daub^2^, Lukas Pichelstorfer^3^, Pontus Roldin^4^*^,^*^5^, Olga Garmash^1,6^, Prasenjit Seal^1^, Theo Kurtén^2^, Matti Rissanen^1^*^,^*^2∗^

^1^Aerosol Physics Laboratory, Tampere University, FI-33101 Tampere, Finland

^2^Department of Chemistry, University of Helsinki, P.O. Box 55, FI-00014 Helsinki, Finland

^3^Pi-Numerics, 5202 Neumarkt am Wallersee, Austria

^4^Department of Physics, Lund University, P.O. Box 118, SE-221 00 Lund, Sweden

^5^Swedish Environmental Research Institute IVL, SE-211 19 Malmö, Sweden

^6^Department of Atmospheric Sciences, University of Washington Seattle, WA, USA

**∗Corresponding author:**

Siddharth Iyer (siddharth.iyer@tuni.fi)

Matti Rissanen (matti.rissanen@tuni.fi)

Contents

[1 Short residence time experiments 2](#_Toc141612474)

[2 Toluene oxidation scheme 4](#_Toc141612475)

[2.1 Fate of C-alkyl and C-peroxy 5](#_Toc141612476)

[2.2 Loss rate of B-alkyl 8](#_Toc141612477)

[3 Comparison with previous toluene oxidation experiments 10](#_Toc141612478)

[4 NO experiments 12](#_Toc141612479)

[5 D_2_O experiments 14](#_Toc141612480)

[6 Ring breaking rate coefficients for other toluene BPR isomers and other aromatics 16](#_Toc141612481)

[7 Rearrangement reactions of bicyclic radicals and closed-shell products from OH reactions with benzene and toluene 17](#_Toc141612482)

[8 Branching between autoxidation and the formation of closed-shell species 19](#_Toc141612483)

[9 Flow tube simulations 21](#_Toc141612484)

[10 Arrhenius plot of rearrangement reaction of toluene i-BPR 24](#_Toc141612485)

[11 T1 diagnostics and %TAE 24](#_Toc141612486)

[12 Reverse reactions along the red R_3_ pathway 24](#_Toc141612487)

[13 References 25](#_Toc141612488)

# 1 Short residence time experiments

Using an injector tube to add toluene to the reactor, the residence time between toluene and OH was reduced to 1.5 seconds and 0.8 seconds. Supplementary Table 1 shows the mass / charge (Th) of dominant peaks from the OH reaction of toluene and CD_3_-toluene. Supplementary Figures 1, 2 and 3 show the results of the experiments carried out at 0.8, 1.5 and 3.7 second residence times, respectively. The results clearly show the rapid formation of the 9-oxygen containing O9-RO_2_.

Supplementary Table 1- Integer mass / charge (Th) of important peaks from the OH reaction of toluene and CD_3_-toluene.

| Molecule (clustered to NO_3_^-^) | Mass / charge (Th) |
| --- | --- |
| C_7_H_9_O_7_ | 267 |
| C_7_D_3_H_6_O_7_ | 270 |
| C_7_H_9_O_9_ | 299 |
| C_7_D_3_H_6_O_9_ | 302 |
| C_7_H_9_O_11_ | 331 |
| C_7_D_3_H_6_O_11_ | 334 |


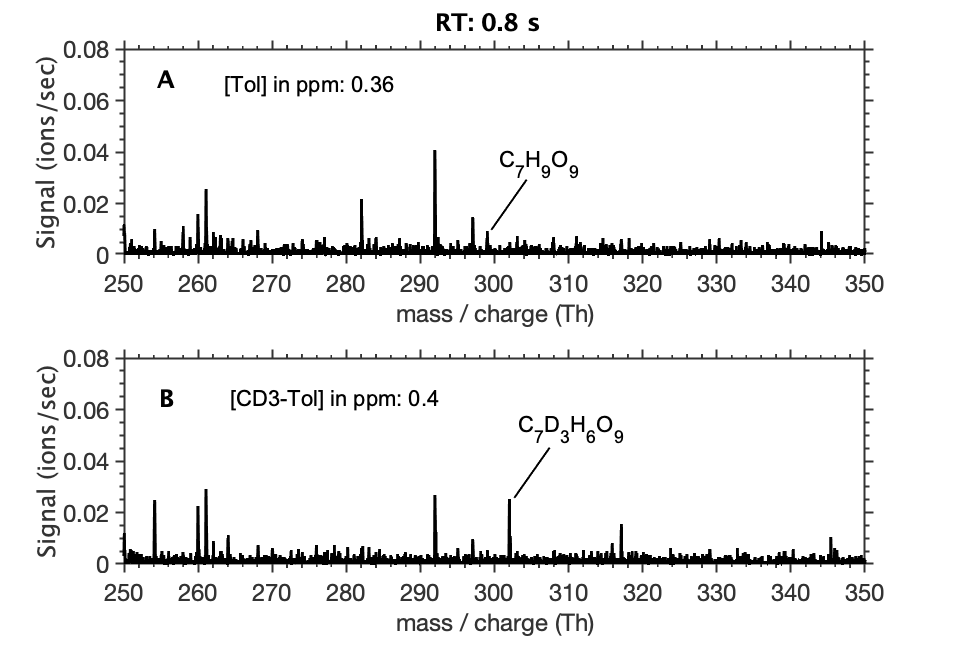


Supplementary Figure 1 – Mass spectra of experiment where OH reacts with (A) toluene (C_7_H_8_) and (B) CD_3_-toluene (C_7_D_3_H_6_) with a residence time of 0.8 seconds. [Toluene]: 0.36 ppm, [CD_3_-toluene]: 0.4 ppm, [TME]: 0.04 ppm, [O_3_]: 0.2 ppm.


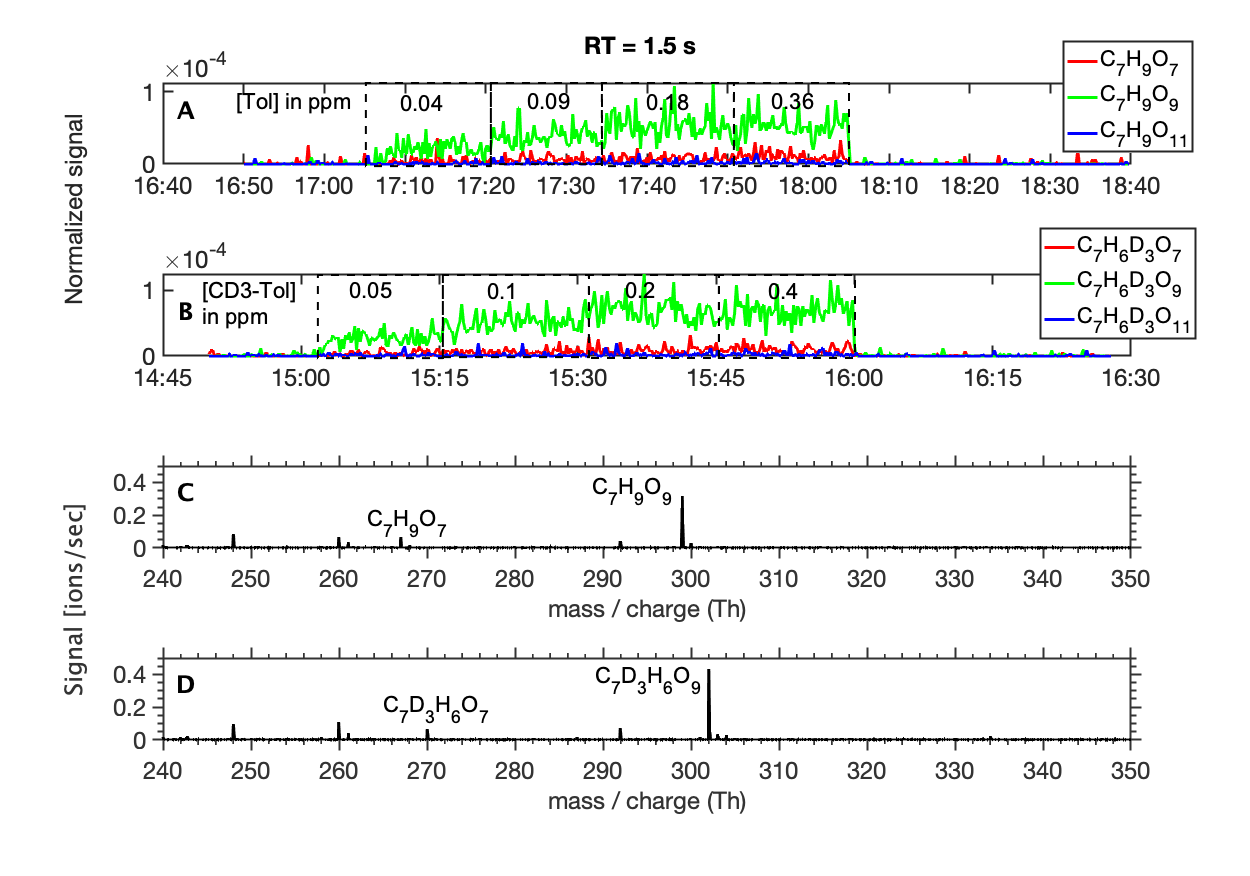


Supplementary Figure 2 - Product distribution (A,B) and mass spectra (C,D) of experiments where OH reacts with toluene and CD_3_-toluene, respectively, with a residence time of 1.5 seconds. Product signals reducing to near zero corresponds to 0 toluene/CD_3_-toluene flow. [TME]: 0.04 ppm, [O_3_]: 0.2 ppm.


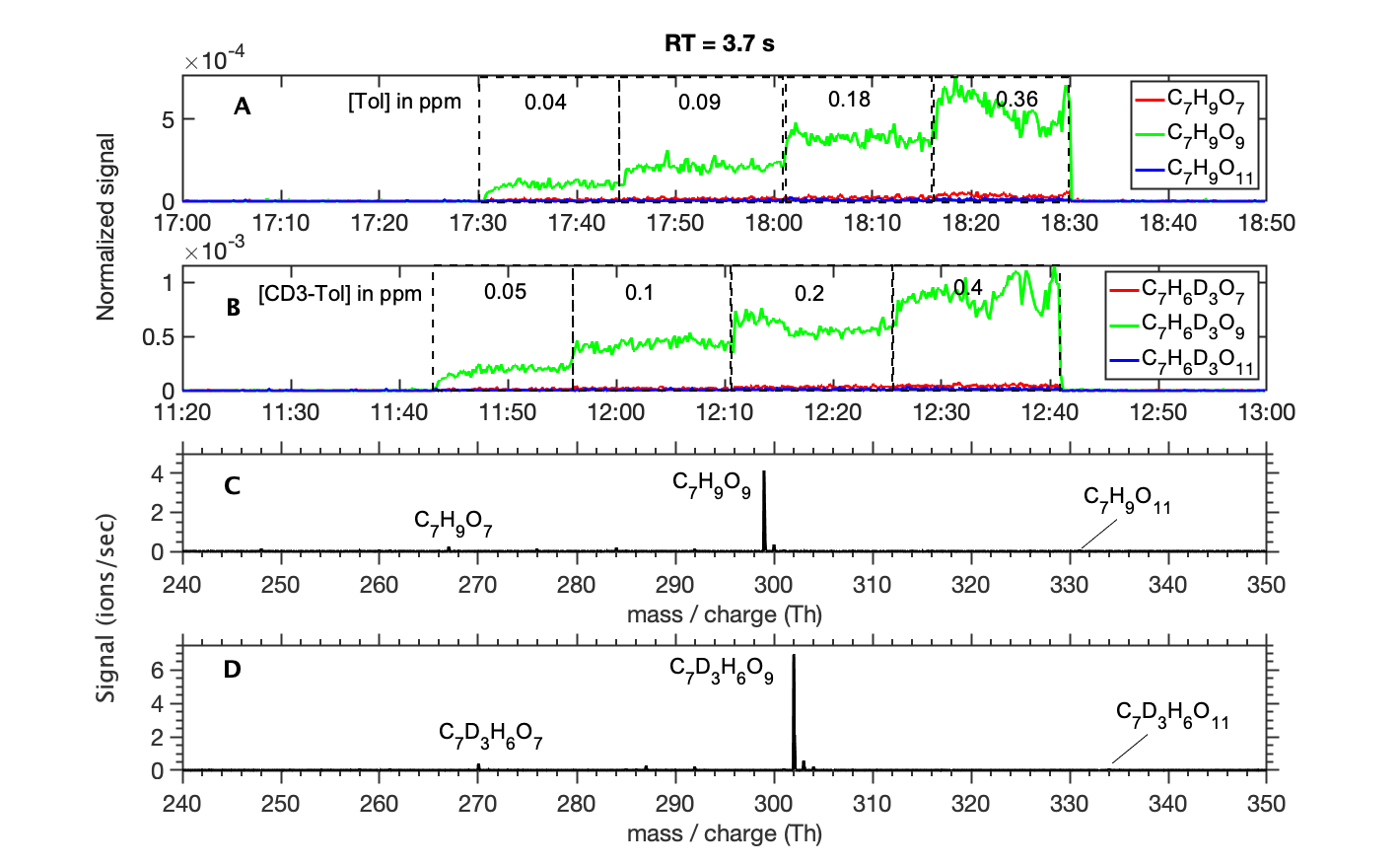


Supplementary Figure 3 - Product distribution (A,B) and mass spectra (C,D) of experiments where OH reacts with toluene and CD_3_-toluene, respectively, with a residence time of 3.7 seconds. Product signals reducing to near zero corresponds to 0 toluene/CD_3_-toluene flow. [TME]: 0.05 ppm, [O_3_]: 0.23 ppm.

# 2 Toluene oxidation scheme

Toluene is one of the most abundant aromatic compounds in the atmosphere, and its oxidation plays an important role in the formation of tropospheric ozone and secondary organic aerosol (SOA). The initial steps of OH addition to toluene is shown in Supplementary Figure 4 and have been discussed in detail in previous works (*1-4*). While OH additions to non-ipso positions can lead to cresol, addition to the ipso position is followed primarily by O_2_ addition to form a peroxy radical. The fates of the peroxy radical and the subsequent intermediates are discussed next. Previously, the mechanistic understanding of the fate of BPR was primarily bimolecular reactions with e.g., NO and HO_2_ because of slow unimolecular rates. This work reports a new unimolecular pathway available to BPR with a competitive reaction rate coefficient that leads to highly oxygenated SOA precursor molecules sub-second timescales.


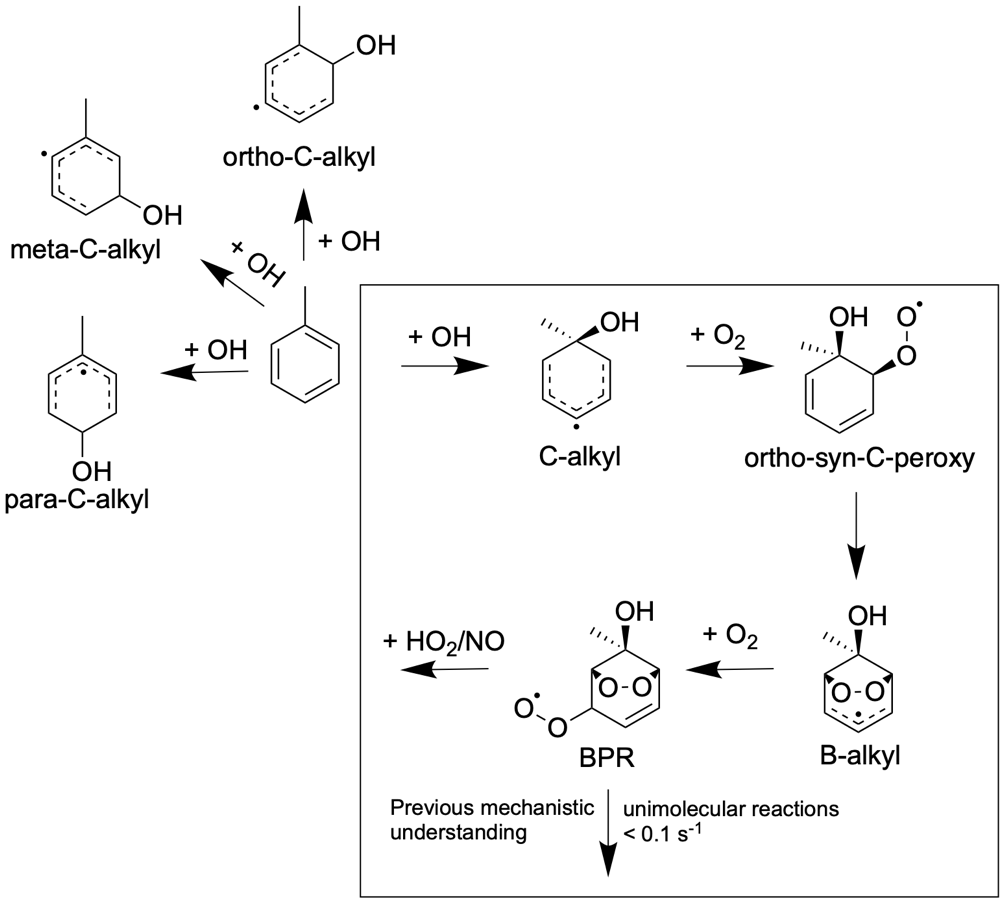


Supplementary Figure 4 – OH initiated oxidation of toluene by addition reaction. The critical early steps are detailed for the OH addition to the ipso site (inside box). Previous mechanistic understanding was that the unimolecular reactions of BPR are too slow to compete with bimolecular reactions with trace atmospheric radicals.

## 2.1 Fate of C-alkyl and C-peroxy

Under atmospheric conditions, the primary fate of C-alkyl is to add O_2_ to form a peroxy radical. The O_2_ addition is site specific, and addition to the para position has been calculated to be faster than ortho(*5*), however the subsequent reactions of para-C-peroxy, with O_2_ in both anti and syn positions relative to OH, are slow (see Supplementary Figure 5 and Supplementary Table 2). Note that B3LYP/6-31+G(d) is a relatively low level of theory. Because the unimolecular reactions of ortho-C-peroxy are calculated to be orders of magnitude faster (see Supplementary Tables 3 and 4), ortho O_2_ addition channels likely funnel the bulk of the formation of highly oxygenated products.


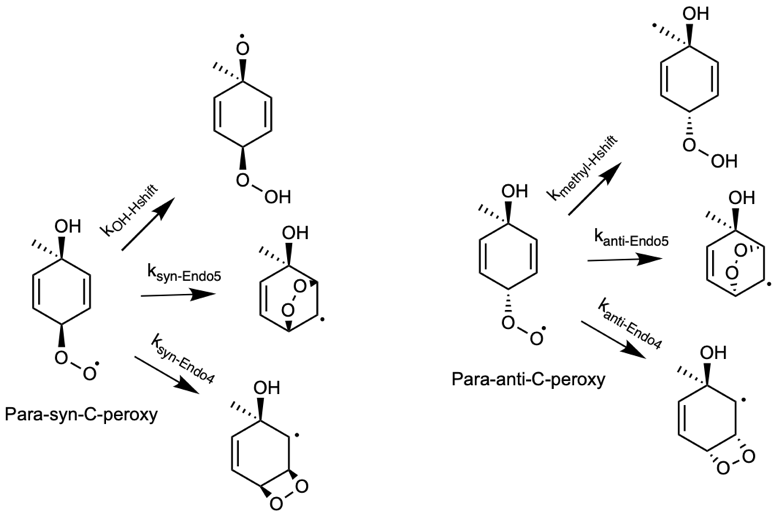


Supplementary Figure 5 - Unimolecular reactions of toluene + OH derived ipso para-C-peroxy with O_2_ in the syn and anti positions relative to OH.

Supplementary Table 2 - Calculated unimolecular rate coefficients using MC-TST method at B3LYP/6-31+G(d) level of theory.

| Para-C-peroxy |  |  |  |
| --- | --- | --- | --- |
| syn | Rate (s^-1^) | anti | Rate (s^-1^) |
| OH-Hshift | 1.3×10^-9^ | Methyl-Hshift | 1.4×10^-14^ |
| Endo4 | 7.0×10^-10^ | Endo4 | 2.2×10^-10^ |
| Endo5 | 1.8×10^-11^ | Endo5 | 5.1×10^-13^ |

The O_2_ addition to the ortho position also produces syn and anti isomers. However, the former is known to dominate due to a lower energy barrier for O_2_ addition and for the subsequent cyclization(*5*). This corroborates with the energy barriers for the syn and anti O_2_ additions we calculated at the F12 level of theory: 2.5 kcal/mol and 3.4 kcal/mol, respectively. Nevertheless, if formed, the potential fates of ortho-anti-C-peroxy is shown in Supplementary Figure 6 with the calculated rate coefficients shown in Supplementary Table 3. Because of issues with F12 calculations on H-abstractions from -OH groups (see Methods), the OH H-shift rate coefficient was computed at the ωB97X-D/aug-cc-pVTZ level of theory. The unimolecular rate coefficients of ortho-syn-C-peroxy, specifically endo-cyclization Endo5-C6 and OH-Hshift, are significantly faster than ortho-anti-C-peroxy (see Supplementary Figure 7 and Supplementary Table 4). Therefore, forming B-alkyl via ortho-syn-C-peroxy is likely the dominant fate of ipso OH addition to toluene.


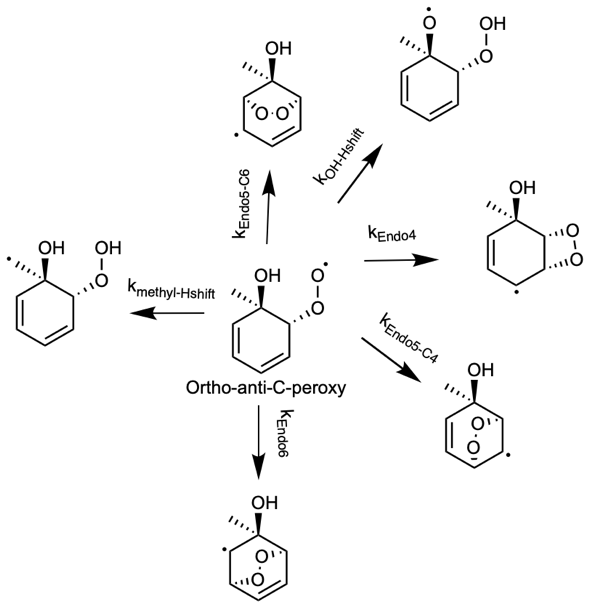


Supplementary Figure 6 - Unimolecular reactions of Ortho-anti-C-peroxy.

Supplementary Table 3 - Unimolecular rate coefficients calculated using MC-TST at ^a^F12, ^b^ωB97X-D/aug-cc-pVTZ and ^c^B3LYP/6-31+G(d) levels of theory.

| Ortho-anti-C-Peroxy | Rate (s^-1^) |
| --- | --- |
| Endo5-C6 | 2.2×10^-1 a^ |
| OH-Hshift | 3.7×10^-3 b^ |
| Endo4 | 8.2×10^-4 c^ |
| Endo5-C4 | 2.2×10^-12 c^ |
| Endo6 | 1.9×10^-7 c^ |
| Methyl-Hshift | 4.9×10^-7 c^ |


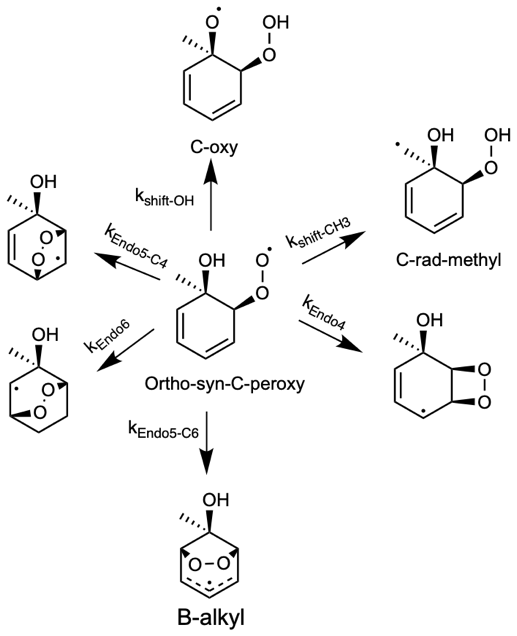


Supplementary Figure 7 - Reaction scheme of Ortho-syn-C-peroxy.

Supplementary Table 4 - Unimolecular rate coefficients calculated using MC-TST at ^a^F12, ^b^ωB97X-D/aug-cc-pVTZ and ^c^B3LYP/6-31+G(d) levels of theory.

| Ortho-syn-C-Peroxy | Rate (s^-1^) |
| --- | --- |
| Endo5-C6 | 2.9×10^2 a^ |
| OH-Hshift | 5.8×10^-2 b^ |
| Endo4 | 1.0×10^-2 c^ |
| Endo5-C4 | 4.7×10^-10 c^ |
| Endo6 | 4.1×10^-5 c^ |
| Methy-Hshift | 6.7×10^-8 c^ |

## 2.2 Loss rate of B-alkyl

In addition to adding an O_2_ to form BPR, B-alkyl can undergo an isomerization reaction to form B-oxy (see Supplementary Figure 8 (*2-4*)). This path has been shown to be unimportant for the benzene derived BPR(*6*). To probe the importance of the ipso-BPR channel for toluene, we compare the effective loss rate of B-alkyl via O_2_ addition to form BPR (k_eff,BPR_) and by isomerization to B-oxy (see Supplementary Figure 8). Following a similar procedure as followed by Xu et al.(*6*), k_eff,BPR_ can be calculated by considering the reversible O_2_ addition to B-alkyl, and assuming molecular rearrangement (k_mr_; proposed in this work) as the only fate of BPR:

$k_{eff,BPR}= k_{mr}\frac{k_{add}}{k-add}{[O}_{2}]$= $k_{mr}K_{eq,add}[O_{2}]$ (2)

Where k_mr_ = 0.16 + 0.59 = 0.75 s^-1^. K_eq,add_ is the equilibrium constant = 1.3×10^-15^ calculated at F12 level of theory. Assuming atmospheric O_2_ concentration and by applying these values into equation (2), k_eff,BPR_ is calculated to be 4.9×10^3^ s^-1^. Alternatively, assuming reaction with NO as the sole fate of the BPR (with [NO] = 1 ppbv and k_RO2+NO_ = 9.15×10^-12^), k_eff,BPR_ is calculated to be 1.5×10^3^ s^-1^. On the other hand, the loss rate of B-alkyl by isomerization into B-oxy, k_oxy_, is calculated to be 2.2×10^-1^ s^-1^ at F12 level of theory. Thus, the formation of B-oxy is unlikely to be competitive under atmospheric conditions as k_oxy_ is orders-of-magnitude slower than the formation of BPR.


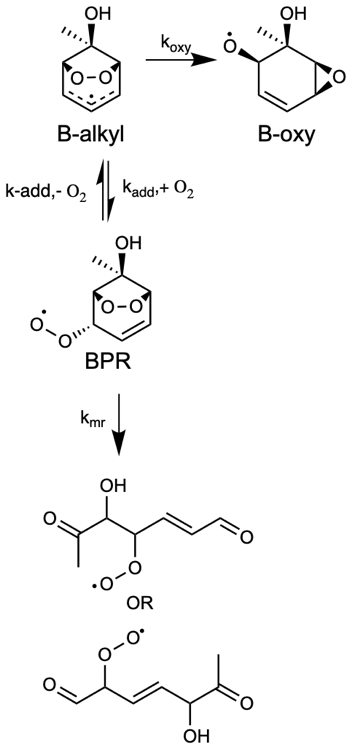


Supplementary Figure 8 - Reaction scheme of B-alkyl.

The ring rearrangement mechanism reported in this work is the first such fate of the BPR reported to date. Supplementary Figure 9 shows the results of the intrinsic reaction coordinate calculation (at ωB97X-D/aug-cc-pVTZ level of theory) on the ring rearrangement mechanism C_2_ transition state for the toluene derived ipso BPR. It shows that the transition state connects to the BPR on the reactant side, and to the completely ring broken RO_2_, P-C_2_, on the product side.


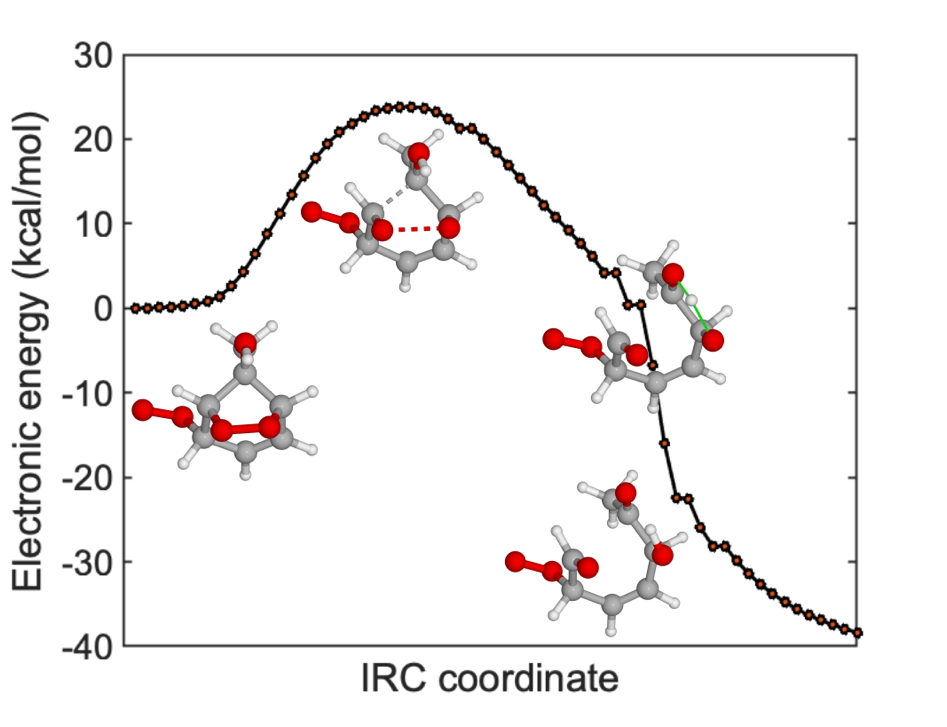


Supplementary Figure 9 – Rearrangement mechanism of toluene i-BPR. Intrinsic reaction coordinate (IRC) calculations on the C_2_ transition state reveals the reaction and product wells. Note that it is electronic energies (kcal/mol) along the y-axis at ωB97X-D/aug-cc-pVTZ level of theory, while it is ROHF-ROCCSD(T)-F12a/VDZ-F12/ωB97X-D/aug-cc-pVTZ zero-point corrected energies in Fig. 1 A in the main manuscript.

# 3 Comparison with previous toluene oxidation experiments

The formation of SOA precursors from toluene oxidation has been extensively studied in the past years(*7-10*). Molteni et al. observed that toluene oxidation produced monomer peaks with up to 11 oxygen atoms(*7*) (Fig. 2 and conclusions in their main manuscript), possibly indicating an autoxidation radical chain reaction occurs that adds several oxygen atoms to the initial BPR. It should be noted that they had high OH concentrations during their experiments, and products derived from multiple OH attacks cannot be discounted. Wang et al. 2017(*11*) showed that the fastest unimolecular reaction of the toluene derived BPR is for OH in the para position and with a rate coefficient of 2.6 × 10^-2^ s^-1^ at 298 K. This corresponds to the abstraction of the H-atom from the methyl group of toluene and leads to the formation of the peroxy radical C_7_H_9_O_7_ following the addition of O_2_. They reported that this peroxy radical rapidly loses an OH (with a rate coefficient of 8 s^-1^) to form the closed-shell species C_7_H_8_O_6_ (see black box in Supplementary Figure 10). To investigate if this is a major pathway in toluene oxidation, we conducted experiments with CD_3_-toluene (see red box in Supplementary Figure 10). The measurement of the O_6_ closed-shell species with one of the deuterated atoms missing would demonstrate the importance of this pathway. However, as can be observed in Supplementary Figure 11, the dominant closed-shell products contained all three D atoms, while lower signals of the corresponding species minus one D atom were measured. In addition, the signals of the closed-shell species were significantly lower than the radical signals in general (compare Supplementary Figure 11 A and B with Supplementary Figure 3 B), which is in line with the autoxidation mechanism proposed in Fig. 2 in the main manuscript where no dominant pathway exists that lead to the formation of the closed-shell species under our experimental conditions. While D-shift reactions are known to be slower than H-shifts, and the CD_3_-toluene experiments could underestimate the role of methyl H-shifts, we also measured low signals of the closed-shell species from the CH_3_-toluene experiments (Supplementary Figure 11 B), indicating that the methyl H-shift is unlikely to play a major role under our experimental conditions.


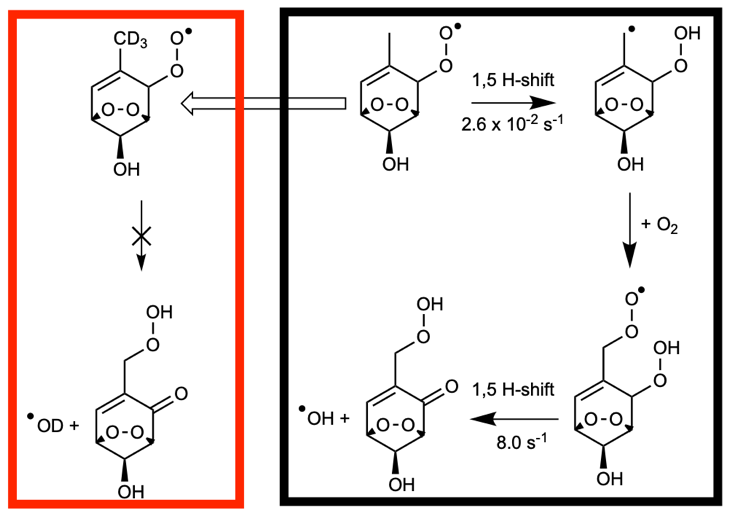


Supplementary Figure 10 – Previous understanding of toluene autoxidation mechanism. Black box: Wang et al. 2017 (11) report that the most competitive unimolecular pathway involves the toluene derived para BPR, and is a 1,5 H-shift from the methyl group. This is followed rapidly by the loss of OH to form the closed-shell molecule C_7_H_8_O_6_. Red box: Our experiments with CD_3_-toluene + OH. The Wang et al. mechanism should lead to an OD loss in our case.


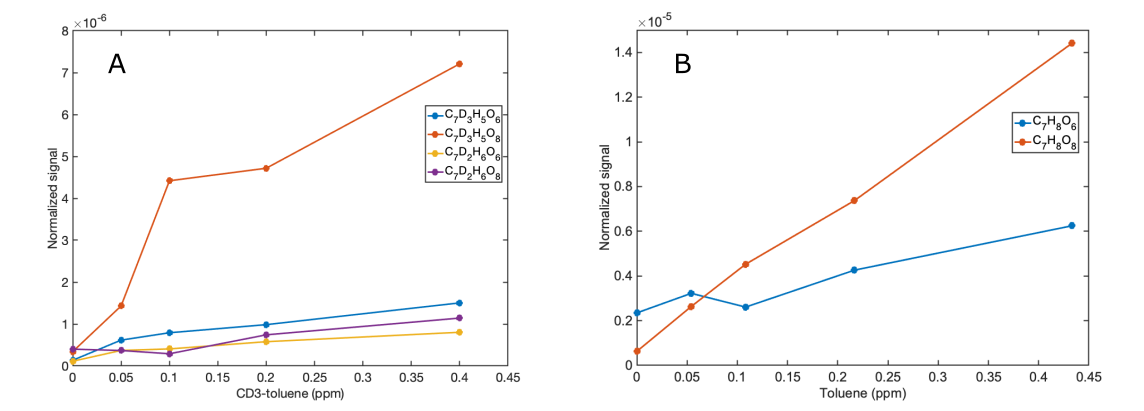


Supplementary Figure 11 – Normalized signal as a function of the concentration of A) CD_3_-toluene and B) toluene of the closed-shell O_6_ and O_8_ products. Signals containing all three deuterated atoms dominate those that have lost one D atom. The closed-shell species are also low in the CH_3_-toluene experiment, indicating that H-shift from the methyl carbon is playing an insignificant role under our experimental conditions.

# 4 NO experiments

Experiments with NO were carried out to test the functionality of the proposed RO_2_ radicals by measuring the corresponding organo nitrates (RO_2_NO). RO_2_NO is formed by the reaction RO_2_ + NO → RONO_2_. Supplementary Table 5 shows the mass / charge (Th) of the important peaks. Supplementary Figure 12 shows the change in product signals and the mass spectra of toluene + OH experiments when NO is added. The detection of the expected organo nitrate peaks and the general decrease of the RO_2_ signals confirm that the signals corresponding to C_7_H_9_O_7_ and C_7_H_9_O_9_ are peroxy radicals. The expected organo nitrate peak of C_7_H_9_O_11_ was also detected. Supplementary Figure 13 is a similar figure from experiments carried out on CD_3_-toluene. These experiments also confirm that the formation of C_7_H_9_O_9_ is rapid since it is detected at non-negligible signals even at high NO concentrations of 0.2 ppm, or 200 ppb, representing an extremely polluted urban air(*12-14*) with corresponding RO_2_ lifetimes around tens of milliseconds.

Supplementary Table 5 - Mass / charge (Th) of important peaks from the OH reaction of toluene and CD_3_-toluene with and without NO.

| Without NO | | With NO | |
| --- | --- | --- | --- |
| Molecule (clustered to NO_3_^-^) | Mass / charge (Th) | Molecule (clustered to NO_3_^-^) | Mass / charge (Th) |
| C_7_H_9_O_7_ | 267 | C_7_H_9_O_7_NO | 297 |
| C_7_D_3_H_6_O_7_ | 270 | C_7_D_3_H_6_O_7_NO | 300 |
| C_7_H_9_O_9_ | 299 | C_7_H_9_O_9_NO | 329 |
| C_7_D_3_H_6_O_9_ | 302 | C_7_D_3_H_6_O_9_NO | 332 |


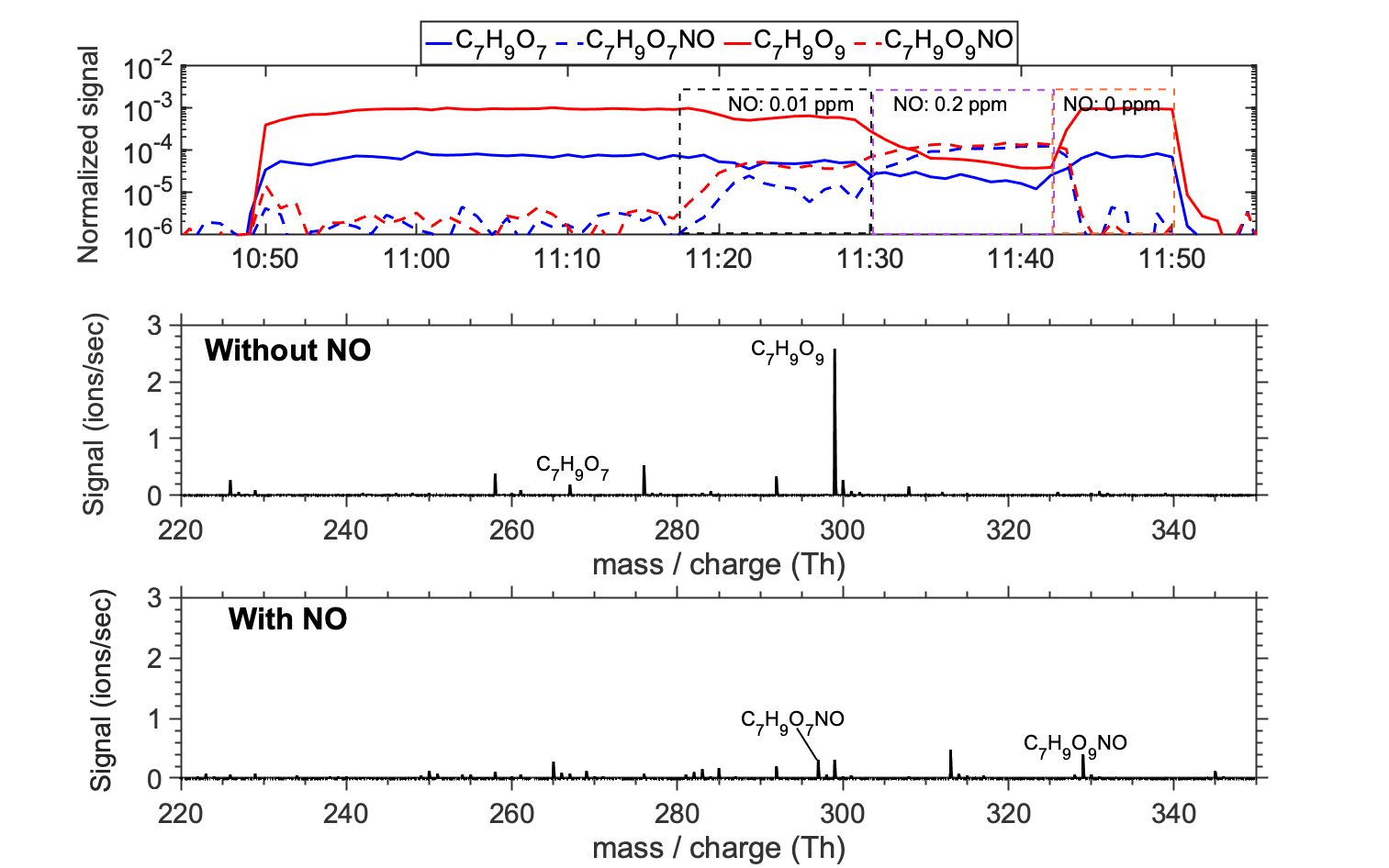


Supplementary Figure 12 – Product distribution of experiments where OH reacts with toluene in the presence and in the absence of NO. Experiment residence time was 3.7 seconds. Top figure shows the variations in the normalized signals of select RO_2_ and RO_2_NO as NO is added to the reactor (1 minute signal averaging time). Middle and bottom figures show the mass spectra in time windows where NO was absent and present, respectively.


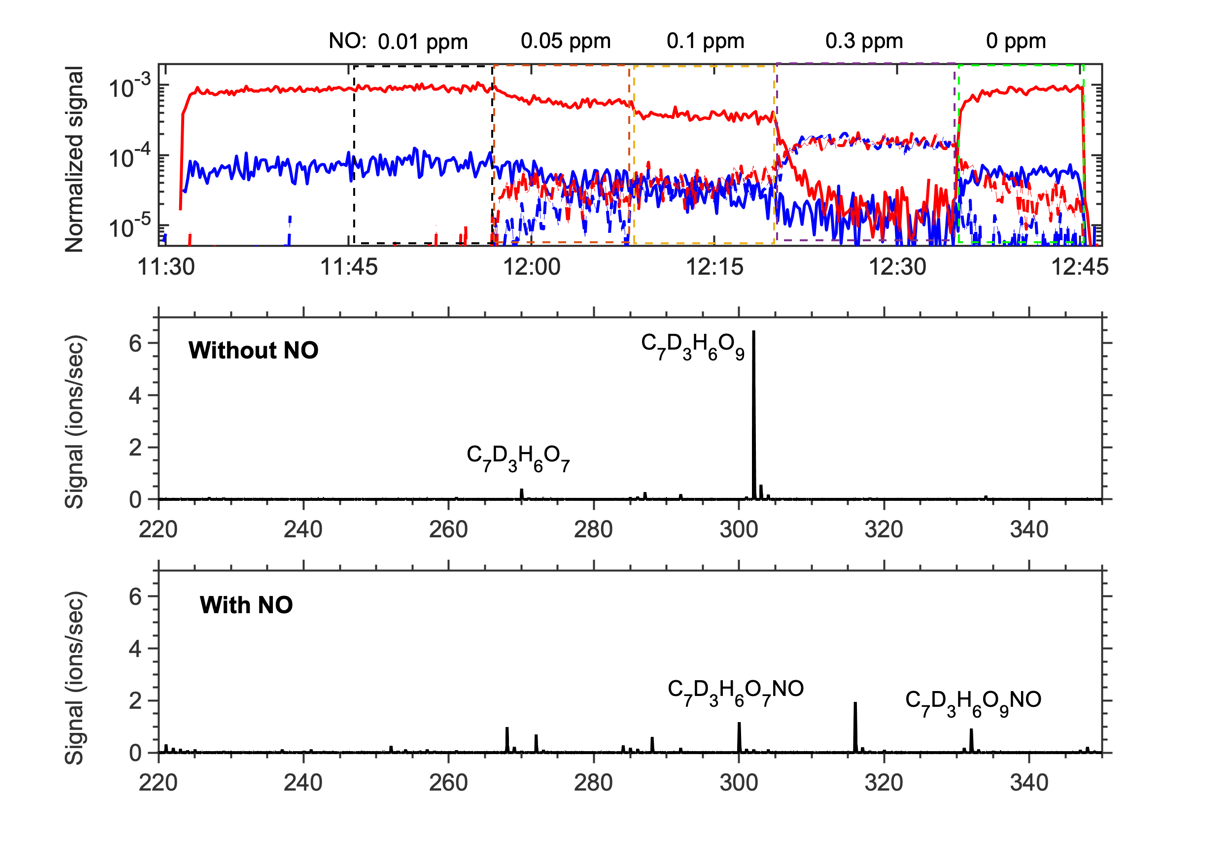


Supplementary Figure 13 – Product distribution of experiments where OH reacts with CD_3_-toluene in the presence and in the absence of NO. Experiment residence time was 3.7 seconds. Time averaging of signals in top figure = 12 seconds.

# 5 D_2_O experiments

Experiments to study the H/D exchange were carried out by adding D_2_O to the flow tube reactor during toluene + OH experiments. These experiments reveal the number of acidic H-atoms in the measured molecules, i.e. the number of -OH and -OOH functional groups. Supplementary Table 6 shows the mass / charge (Th) of the important peaks. As shown in Supplementary Figure 14, these experiments reveal that at least two isomers of the C_7_H_9_O_7_ peroxy radical are detected, one with one acidic functional group and another with two. This corresponds well with the autoxidation mechanism proposed in Fig. 2 in the main manuscript. Similarly, the proposed autoxidation mechanism points to the C_7_H_9_O_9_ peroxy radical isomer with two acidic functional groups that forms rapidly and is likely the dominant signal. This is corroborated by the D_2_O experiment. The corresponding peaks for the CD_3_-toluene experiment is shown in Supplementary Figure 15. The D_2_O experiments also indicate three isomers for the 11-oxygen containing C_7_H_9_O_11_ peroxy radical with two, three and four H->D exchanges. While isomers with three labile H-atoms agrees with the proposed mechanism in Fig. 2, the source of the other two isomers could be autoxidation along the P-C_1_ pathway not explored in this work.

Supplementary Table 6 - Mass / charge (Th) of important peaks from the OH reaction of toluene and CD_3_-toluene with and without D_2_O. The number of H/D exchanges are indicated in parenthesis.

| Molecule (clustered to NO_3_^-^) | Mass / charge (Th) |
| --- | --- |
| C_7_H_9_O_7_ | 267 |
| C_7_D_1_H_8_O_7_ (= +1) | 268 |
| C_7_D_2_H_7_O_7_ (= +2) | 269 |
| C_7_D_3_H_6_O_7_ | 270 |
| C_7_D_4_H_5_O_7_ (= +1) | 271 |
| C_7_D_5_H_4_O_7_ (= +2) | 272 |
| C_7_H_9_O_9_ | 299 |
| C_7_D_2_H_7_O_9_ (= +2) | 301 |
| C_7_D_3_H_6_O_9_ | 302 |
| C_7_D_5_H_4_O_9_ (= +2) | 304 |


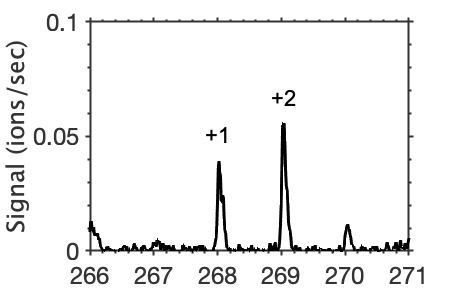

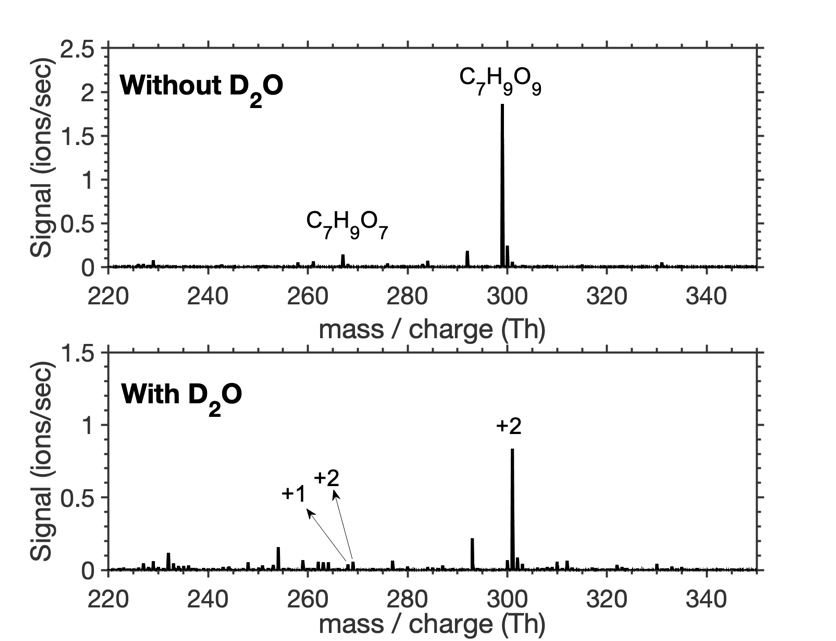


Supplementary Figure 14 – Mass spectra recorded of toluene + OH experiments in the absence (top) and presence of heavy water D_2_O. Experiment residence time was 3.7 seconds. The bottom figure illustrates the signal shift, thereby indicating the number of -OH and -OOH groups in the detected molecule. At least two isomers of C_7_H_9_O_7_ are detected, one with one acidic functional group (+1) and one with two acidic functional groups (+2). C_7_H_9_O_9_ with two acidic functional groups (+2) is the dominant isomer.


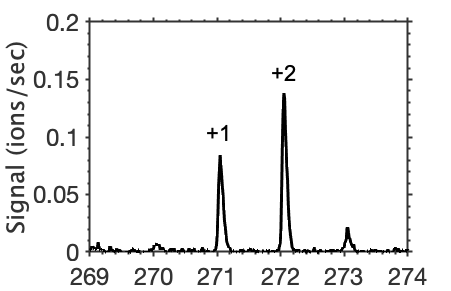

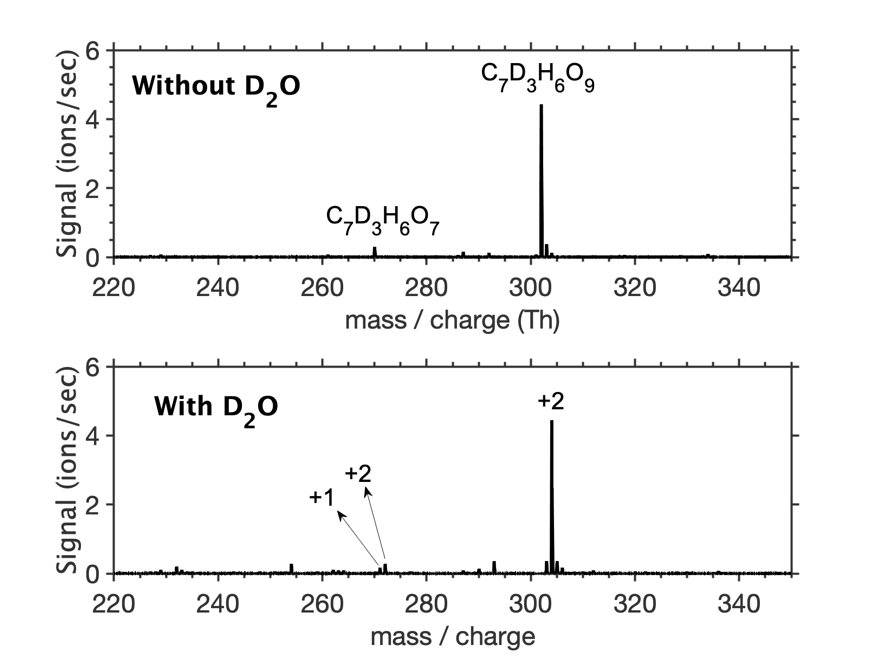


Supplementary Figure 15 - Mass spectra recorded of CD_3_-toluene + OH experiments in the absence (top) and presence of heavy water D_2_O. Experiment residence time was 3.7 seconds.

# 6 Ring breaking rate coefficients for other toluene BPR isomers and other aromatics

The rearrangement mechanism is the fastest for ipso BPR, but also exists for the other BPR isomers. Supplementary Table 7 shows the rate coefficients of the rearrangement mechanism for the four possible BPR isomers of the toluene + OH system.

Supplementary Table 7 – Energy barriers and rate coefficients of ring breaking reactions of the four possible BPR isomers of the toluene + OH system. Of the two possible ring breaking pathways, only the one with the lowest energy barrier is shown.

| OH addition site | Barrier (kcal/mol) | Rate (s^-1^) |
| --- | --- | --- |
| Ipso | 17.9 | 0.59 |
| Meta | 21.0 | 3.0×10^-3^ |
| Para | 22.0 | 1.0×10^-3^ |
| Ortho | 23.2 | 9.0×10^-5^ |

#

# 7 Rearrangement reactions of bicyclic radicals and closed-shell products from OH reactions with benzene and toluene

In addition to BPRs, the rearrangement mechanism described in this work is also possible for other aromatic derived bicyclic molecules. Supplementary Table 8 shows the computed barriers of rearrangement mechanisms for benzene and toluene derived bicyclic alkyl radical, peroxy radical, hydroperoxide and organo nitrate.

Supplementary Table 8 – Barriers to the rearrangement of other bicyclic intermediates and products of benzene and toluene.

| Benzene derived | Rearrangement barrier (kcal/mol) | Toluene derived | Rearrangement barrier (kcal/mol) |
| --- | --- | --- | --- |
|  | 22.4 |  | 18.7 |
|  | 21.5 |  | 17.9 |
|  | 22.1 |  | 18.5 |
|  | 22.7 |  | 19.4 |

The previous mechanistic understanding of aromatic oxidation was that the primary fate of BPRs under polluted conditions is bimolecular reactions with NO to form B-ROONO intermediates that can either decompose into alkoxy radicals or isomerize to form organo nitrates (B-RONO_2_; BPR + NO => B-RONO_2_; B = bicyclic). While only some fraction of BPR + NO reactions will lead to B-RONO_2_, these organo nitrates are thought to be stable once formed. As shown in Supplementary Table 7, the rate coefficients for the molecular rearrangements of non-ipso BPRs from toluene (and for other aromatics; see Fig. 4 in the main manuscript) are likely too slow to be competitive with reactions with NO, and these BPRs will likely lead to a significant fraction of B-RONO_2_s. As described before, these B-RONO_2_s can subsequently undergo molecular rearrangement reactions to form completely ring broken RONO_2_s (RB-RONO_2_). The RB-RONO_2_s can potentially lose the NO_2_ to form ring broken alkoxy radicals (RB-RO) that can further oxidize. Additionally, via this reaction, RONO_2_s could act as transport reservoirs of NO_2_, which has implications for tropospheric ozone formation. Supplementary Figure 16 illustrates the general reaction coordinate for this reaction, and Supplementary Table 9 provides the corresponding zero-point corrected energies (in kcal/mol and at F12 level) and the MESMER derived rate coefficients (in s^-1^) for the key reactions that determine the fate of the RONO_2_s. MESMER simulations indicate that even when the B-ROONO intermediates are neglected, excess energy from RO_2_ + NO => B-RONO_2_ step does not affect the B-RONO_2_ dissociation rates: the dissociation reactions are fully thermal.

The rate coefficient for the conversion of ortho-B-RONO_2_ to ortho-RB-RO + NO_2_ is much too slow to be significant, but the others convert at atmospherically relevant time scales. The conversion of the Tol-ipso-B-RONO_2_ is the fastest, but the BPR is likely to undergo molecular rearrangement as a peroxy radical before reacting with NO to form the organo nitrate. The relatively fast conversion of the benzene derived B-RONO_2_ to RB-RO + NO_2_ is significant as it corresponds to an atmospheric lifetime of Ben-B-RONO_2_ of about 34 minutes. Similarly, the atmospheric lifetimes of Tol-meta-B-RONO_2_ and Tol-para-B-RONO_2_ are about 28 minutes and 3 hours, respectively.


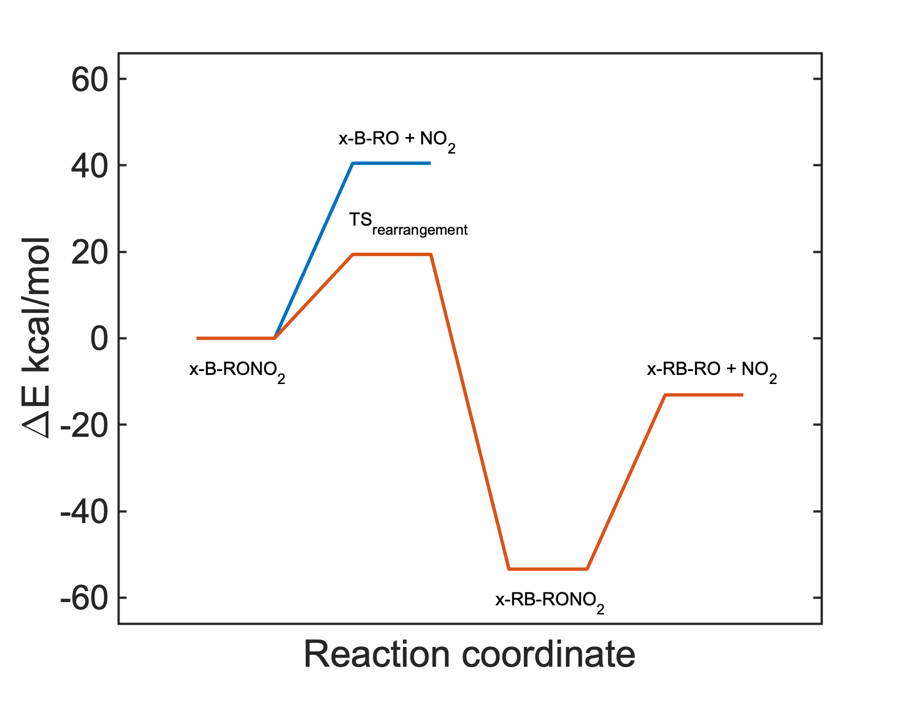


Supplementary Figure 16 – General reaction coordinate of BPR + NO reaction. Note that BPR + NO will first lead to a ROONO intermediate and only a fraction will subsequently isomerize to the more stable RONO_2_ isomer (the rest directly decomposing into RO + NO_2_). We only include RONO_2_ here as we are interested in the fate of the intermediate that is generally considered to be stable under atmospheric conditions. Here, x = benzene and toluene derived ipso, ortho, meta and para. B = bicyclic, RB = ring broken.

Supplementary Table 9 - Relative energies (in kcal/mol) and MESMER derived rate coefficients (in s^-1^) corresponding to the stationary points in  Supplementary Figure 16. Here, x = benzene and toluene derived ipso, ortho, meta and para. B = bicyclic, RB = ring broken.

|  | Benzene | Toluene | | | |
| --- | --- | --- | --- | --- | --- |
|  |  | Ipso | Ortho | Meta | Para |
| x-B-RONO_2_ | 0 | 0 | 0 | 0 | 0 |
| x-B-RO + NO_2_ | 41.1 | 40.5 | 41.1 | 38.4 | 41 |
| TS_rearrangement_ | 22.2 | 19.4 | 25.5 | 21.2 | 21.9 |
| x-RB-RONO_2_ | -48.8 | -53.4 | -51.2 | -46 | -42.5 |
| x-RB-RO + NO_2_ | -6.5 | -13.1 | -9.3 | -5.7 | -0.3 |
| k_x-B-RONO2 => x-B-RO + NO2_ | 9.3×10^-12^ | 1.6×10^-11^ | 1.3×10^-13^ | 1×10^-9^ | 7.6×10^-12^ |
| k_x-B-RONO2 => x-RB-RO + NO2_ | 4.9×10^-4^ | 0.1 | 3.6×10^-8^ | 6×10^-4^ | 1×10^-4^ |

# 8 Branching between autoxidation and the formation of closed-shell species

Due to site specific O_2_ addition, the alkyl radical R_1_ in Fig. 2 can either lead to the closed-shell species C_7_H_8_O_5_ or continue the autoxidation chain by forming R_1b_-RO_2_ as illustrated in Supplementary Figure 17. The branching fraction of the latter is key to establishing the importance of this channel in the formation of the peroxy radical C_7_H_9_O_9_. Transition state calculations followed by intrinsic reaction coordinate calculations indicated that the H-abstraction by O_2_ to form C_7_H_8_O_5_ is not direct but follows the initial addition of O_2_ to the C(OH) carbon. The yield of R_1b_-RO_2_ is therefore sensitive to the O_2_ addition transition states TS_i_ and TS_j_ forming R_1a_-RO_2_ and R_1b_-RO_2_, respectively. These transition states were calculated to be -0.7 kcal/mol and 1.9 kcal/mol, respectively, relative to R_1_ at F12 level of theory. The formation of R_1_ is significantly exothermic, and excess energy likely plays an important role in the branching fraction of R_1a_-RO_2_ and R_1b_-RO_2_. Therefore, the potential energy surface shown in Supplementary Figure 18 was used as input in a MESMER simulation to account for the excess energy of R_1_. The formation of all intermediates in Supplementary Figure 18 are preceded by transition states and these reactions were treated using the SimpleRRKM method with Eckart tunneling. The intermediates P-C_2_ and R_1_ were treated as “modelled” and R_1a_-RO_2_ and R_1b_-RO_2_ as sinks in the simulation. The MESMER input file is provided in the data archive. This resulted in a 20% yield for R_1b_-RO_2_. Note that the F12 correction causes TS_i_ to be slightly negative, and while this does not impede the MESMER simulations, it does add some uncertainty to the predicted branching ratios. Also, the two O_2_ addition reactions are reversible, which is especially important for R_1a_-RO_2_ given that its formation is less exothermic and R_1_ contains excess energy. We accounted for the reverse reactions of R_1a_-RO_2_ and R_1b_-RO_2_ by including a RO_2_ + NO => ROONO channel with a loss rate of 1 s^-1^. This was done by adding a model ROONO sink to R_1a_-RO_2_ and R_1b_-RO_2_ using the SimpleBimolecularSink method in MESMER with NO concentration of 5×10^10^ molecules/cm^3^ and a reaction rate coefficient of 2×10^-11^ cm^3^/molecule/s. R_1a_-RO_2_ and R_1b_-RO_2_ were treated as “modelled” to account for their reverse reactions. If no sink reactions are added and R_1a_-RO_2_ and R_1b_-RO_2_ are simply treated as “modelled” to account for reverse reactions, then the product that is thermodynamically the most stable is formed with a 100% yield. Therefore, a sink with an approximately correct timescale to correctly model the effect of reversibility is needed. The precise details of the sink do not matter as long as the timescale is correct. Therefore, a model ROONO system computed at the B3LYP/6-31+G(d) level was used with an effective rate of 1 s^-1^. The MESMER input PES is shown in Supplementary Figure 19 and the input file is provided in the data archive. This resulted in 100% yield of R_1b_-ROONO, indicating a larger yield of R_1b_-RO_2_ when low exothermicity of R_1a_-RO_2_ formation is accounted for.


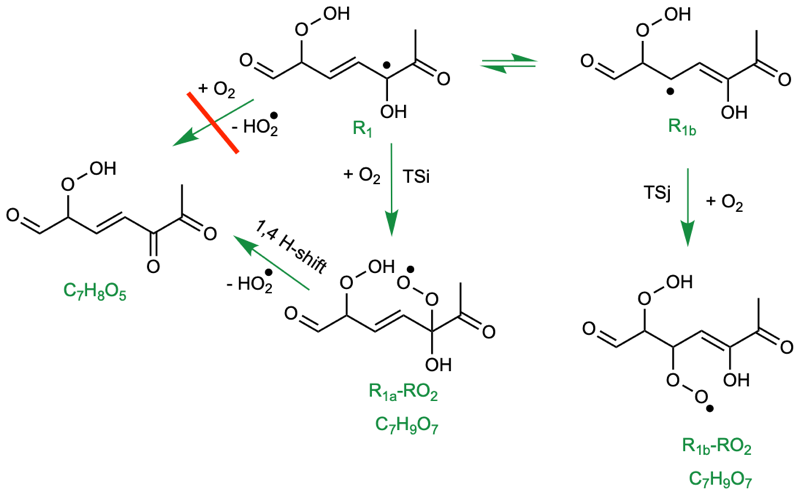


Supplementary Figure 17 – Subsequent reactions of the alkyl radical R_1_. Intrinsic reaction coordinate calculations show that R_1_ does not directly react with O_2_ to form C_7_H_8_O_5_, but first adds O_2_ to form R_1a_-RO_2_ instead. Alternatively, the O_2_ can add to a different carbon to form R1b-RO_2_. The latter is key for the formation of the highly oxygenated O_9_-RO_2_, C_7_H_9_O_9_.


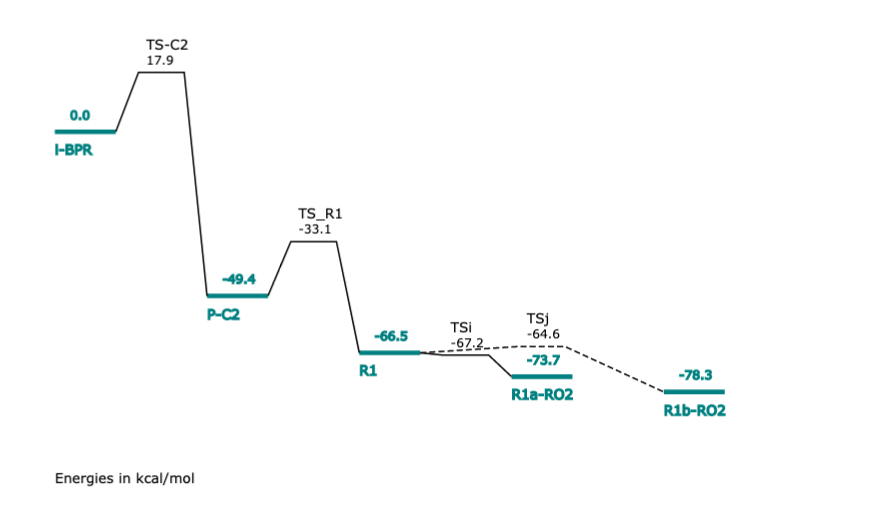


Supplementary Figure 18 – The potential energy surface used in the MESMER simulation to calculate the branching ratios of R_1a_-RO_2_ and R_1b_-RO_2_.


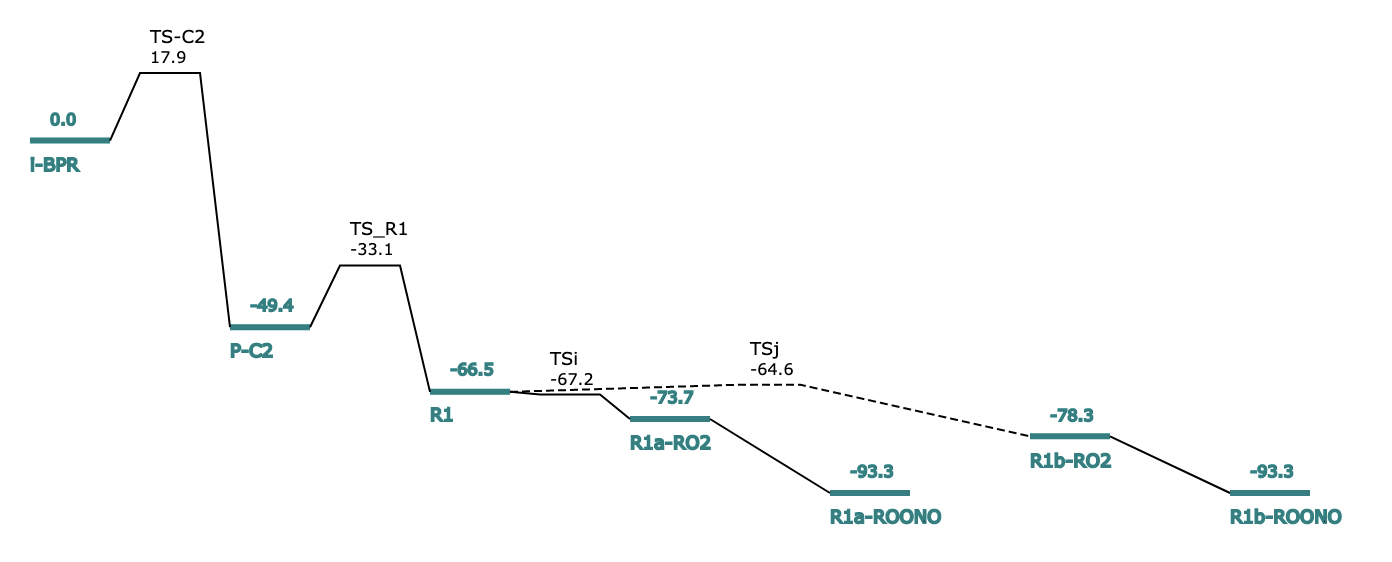


Supplementary Figure 19 - The potential energy surface used in the MESMER simulation to account for the reversibility of O_2_ R_1a_-RO_2_ and R_1b_-RO_2_.

# 9 Flow tube simulations

The chemical system in the simulations is described by the Chemical Mechanism, MCM v3.3.1(*15-17*) including the precursors toluene and TME. The ipso BPR pathway described in this work is embedded into the existing equations. To quantify potential loss of chemical species at the tube walls, a wall loss function was added. It considers the diffusional losses of chemical species in a fully developed laminar flow field(*18*). To allow accounting for local sources/sinks of the species, the analytical solution for diffusion of chemical species in a laminar flow field of the tube is represented by its Taylor expansion(*19*). The range of deposition is considered to lie between zero deposition and a cross section averaged deposition to the walls with a sticking coefficient of 1. The mean deposition is represented by a 50% reduced loss with regard to the formulations suggested by Ingham(*18*) as the reactive species are added at the center line of the flow tube to limit influence of the tube walls. As noted in the manuscript, the computed yields of R_1_, R_2_ and R_3_ in Fig. 2 likely have high uncertainties. Therefore, these were varied in the model and checked for closure with the measured mass spectrum. Of the 79% yield of P-C_2_, the branching of R_1_, R_2_ and R_3_ that reproduced the measurements the best was 92:3:5, respectively. As discussed in the previous section, computations indicate that the branching of R_1_ towards the closed-shell species C_7_H_8_O_5_ is favored. In the model, 85% yield of C_7_H_8_O_5_ and a 15% yield R_1b_-RO_2_ was used. Finally, in the simulations, the RO_2_ + RO_2_ reaction rate coefficients need to be ~1×10^-11^ cm^3^ molec^-1^ s^-1^ or less in order for the model to not overestimate the closed shell products in the flow tube setup.

**
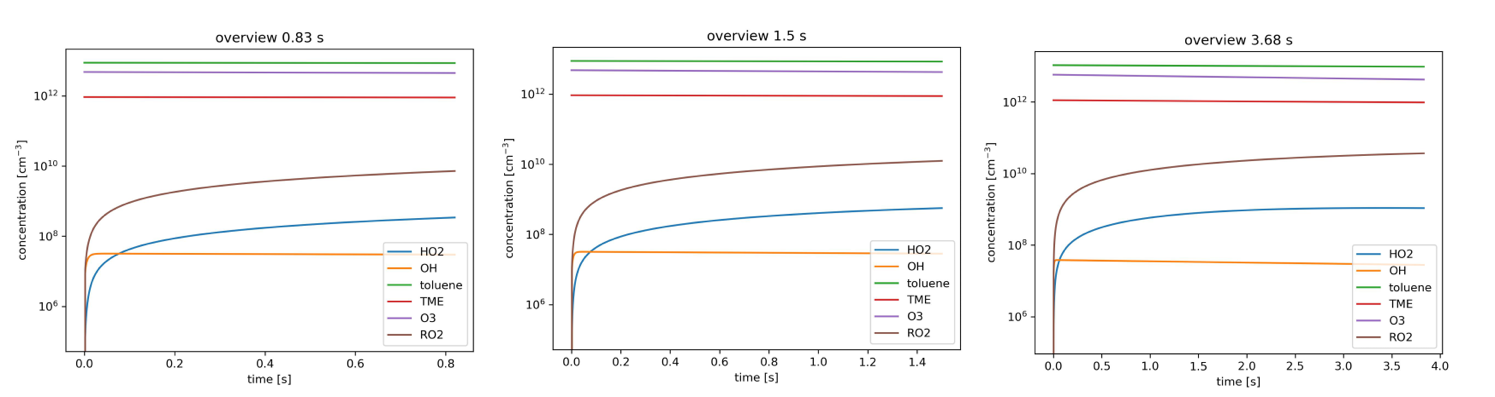
**

Supplementary Figure 20 - Concentrations of OH, RO_2_ and other key species during simulations at different flow tube reaction times. RO_2_ in the figure contains contributions from MCM and from the extended new mechanism of this work.


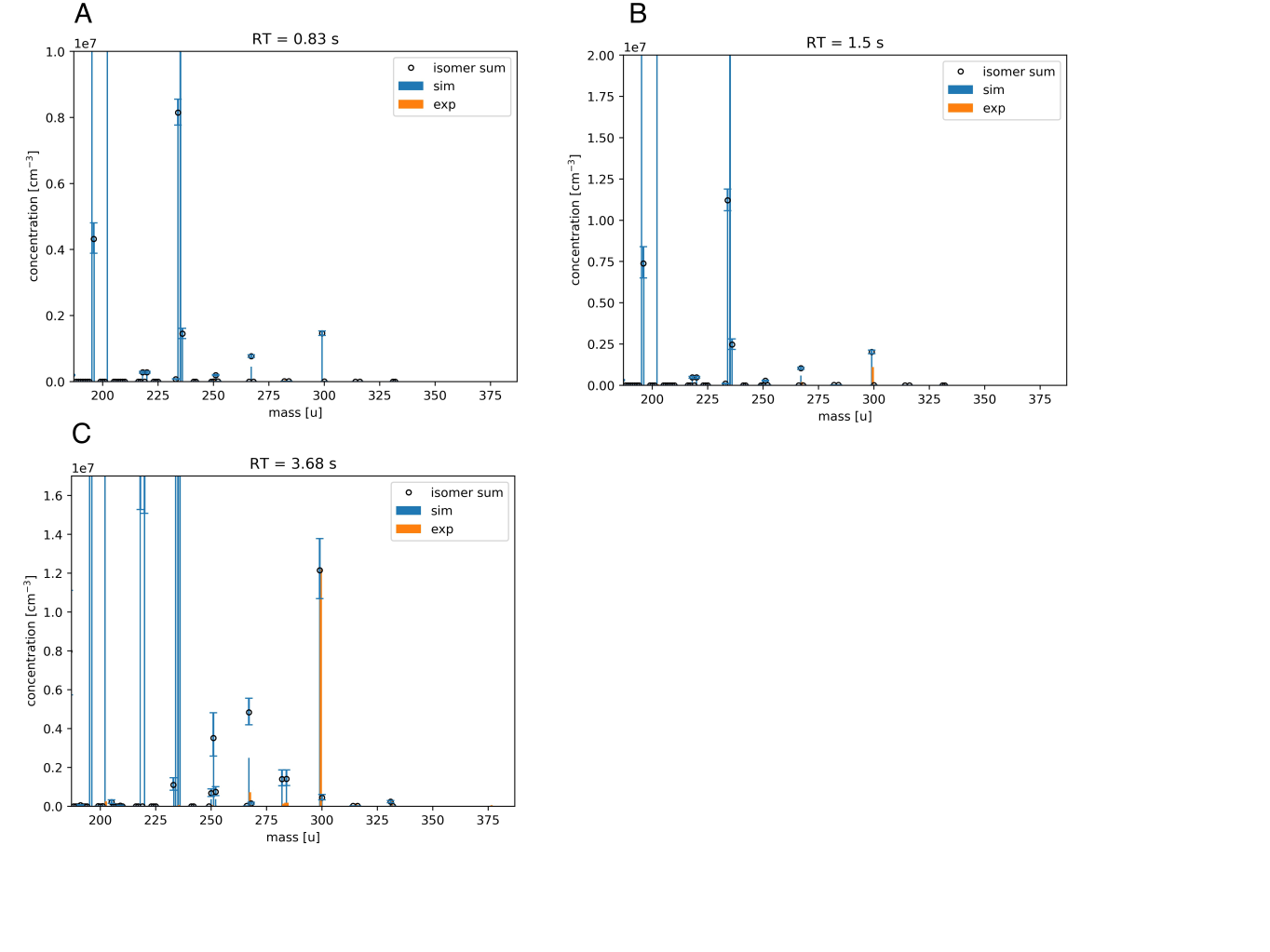


Supplementary Figure 21 - Comparison of experimental findings and simulated results. A) Reaction time in flow tube = 0.83 s, B) RT = 1.5 s and C) RT = 3.68 s. Mass in atomic mass is plotted on the x-axis and y-axis denotes the concentration of the chemical species. While isomers are summed up in the experimental data (yellow bars), the simulation results resolve several isomers (blue bars). The sum of simulated isomers (“isomer sum”) are depicted by black circles. Potential impact by wall losses is illustrated by error bars. The less oxygenated products at smaller atomic masses are not detected in experiments due to the low of sensitivity of the NO_3_-CIMS method for these molecules.

Supplementary Table 10 – Model inputs.

| Simulation time [s] | 0.83 | 1.5 | 3.83 |
| --- | --- | --- | --- |
| C_0_(TME) [cm^-3^] | 9.25×10^11^ | 9.25×10^11^ | 1.12×1012 |
| C_0_(Toluene) [cm^-3^] | 8.8×10^12^ | 8.8d×10^12^ | 1.07×10^13^ |
| C_0_(O_3_) [cm^-3^] | 4.79×10^12^ | 4.79×10^12^ | 5.79×10^12^ |
| T [K] | 298 | 298 | 298 |
| RH [%] | 0.1 | 0.1 | 0.1 |
| Tube diameter [cm] | 4.7 | 4.7 | 4.7 |
| Δt – simulation [s] | 1.0×10^-3^ | 1.0×10^-3^ | 1.0×10^-3^ |

# 10 Arrhenius plot of rearrangement reaction of toluene i-BPR


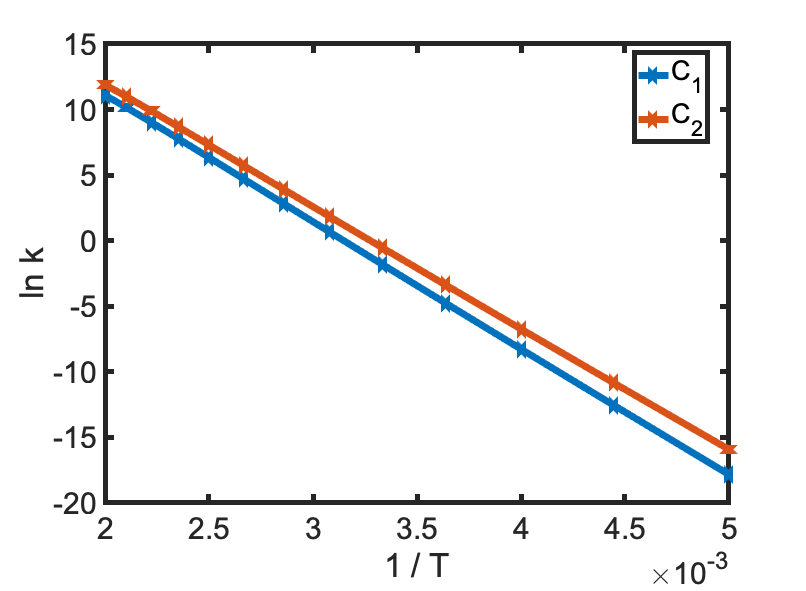


Supplementary Figure 22 - Master equation derived rate coefficients of the rearrangement mechanisms C_1_ and C_2_ as a function of temperature at P = 1 atm.

# 11 T1 diagnostics and %TAE

The reliability of the single reference method used for the molecular rearrangement mechanism was checked by looking at the T1 diagnostic values computed at the CCSD(T)-F12 stage. The values for the toluene derived i-BPR, and transition states T-C_1_ and T-C_2_ are provided below. These are within the threshold values for open-shell systems, indicating that single-reference methods are reliable for this reaction. The T1 values for the other studied aromatics and for the closed-shell species can be extracted from the Molpro output files in the data archive that is made available along with this manuscript.

Supplementary Table 11 - T1 diagnostic values.

| Toluene derived | T1 diagnostic |
| --- | --- |
| i-BPR | 0.02073698 |
| T-C_1_ | 0.02473019 |
| T-C_2_ | 0.02428866 |

Additionally, the triples contribution to the total atomization energy %TAE(T) value for T-C_1_ was found to be 2.7%.

# 12 Reverse reactions along the red R_3_ pathway

The reversibility of R_3_-Epo-R’ and the H-migration from -OOH to -O radical, which is essentially the reverse reaction of R_3_ back to P-C_2_ were checked to get a better estimate of the higher oxygenated peroxy radicals from the red pathway in Fig. 2 in the main manuscript. This was done by running a MESMER simulation along the R_1_, R_2_ and R_3_ pathways of P-C_2_ with parameters calculated at the ωB97X-D/aug-cc-pVTZ level. The lower level of theory is to use the same methodology as that used for computing the TS to R_3_ for which CCSD(T)-F12 calculations are unreliable. For R_3_, we ignored the closed-shell C_7_H_8_O_5_ forming channel and assigned the formation of R_1b_-RO_2_ as the direct sink of R_1_ (+ O_2_). The input PES is shown in Supplementary Figure 23. The simulation shows that R_3_-Epo-RO_2_ is a significant product (36% yield). While the reverse reactions are fast, the excess energy of P-C_2_ likely drives the formation of the R_3_-Epo-R’O_2_.


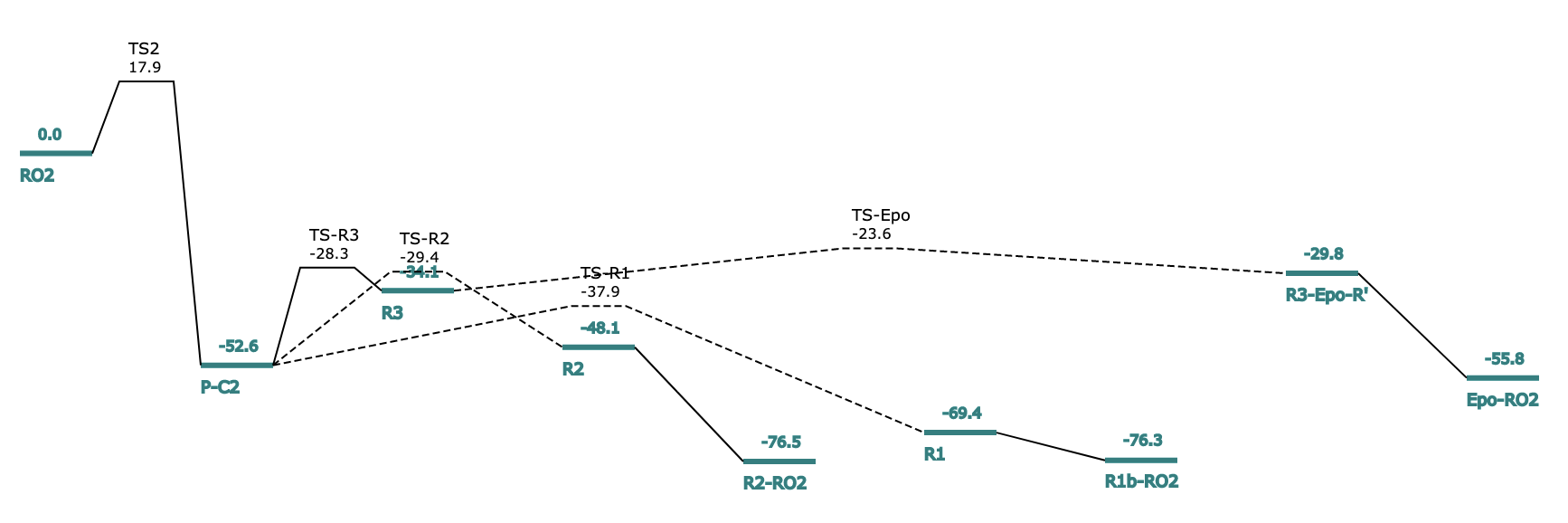


Supplementary Figure 23 - The potential energy surface used in the MESMER simulation to account for the reversibility of R_3_ and R_3_-Epo-’.

# 13 References

1. Calvert, J. G., Atkinson, R., Becker, K. H., Kamens, R. M., Seinfeld, J. H., Wallington, T. J., and Yarwood, G.: The mechanisms of atmospheric oxidation of aromatic hydrocarbons, *Oxford University Press, Oxford, New York*, 566 pp., (2002).
2. Glowacki, D.R., Wang, L. and Pilling, M.J. Evidence of formation of bicyclic species in the early stages of atmospheric benzene oxidation. *The Journal of Physical Chemistry A*, *113*(18), pp.5385-5396 (2009).
3. Vereecken, L. and Francisco, J.S. Theoretical studies of atmospheric reaction mechanisms in the troposphere. *Chemical Society Reviews*, *41*(19), pp.6259-6293 (2012).
4. Vereecken, L.: Reaction mechanisms for the atmospheric oxidation of monocyclic aromatic compounds, in: Advances in Atmospheric Chemistry: Volume 2: Organic Oxidation and Multiphase Chemistry, edited by: Barker, J. R., Steiner, A. L., and Wallington, T. J., World Scientific Publishing Co. Pte. Ltd., Singapore, 377–527, <https://doi.org/10.1142/9789813271838_0006>, (2019).
5. R. Wu, S. Pan, Y. Li, L. Wang, Atmospheric oxidation mechanism of toluene. *The Journal of Physical Chemistry A* 118, 4533–4547 (2014).
6. L. Xu, K. H. Møller, J. D. Crounse, H. G. Kjaergaard, P. O. Wennberg, New insights into the radical chemistry and product distribution in the OH-initiated oxidation of benzene. *Environmental Science & Technology* 54, 13467–13477 (2020).
7. U. Molteni, F. Bianchi, F. Klein, I. El Haddad, C. Frege, M. J. Rossi, J. Dommen, U. Baltensperger, Formation of highly oxygenated organic molecules from aromatic compounds. *Atmospheric Chemistry and Physics* 18, 1909–1921 (2018).
8. O. Garmash, M. P. Rissanen, I. Pullinen, S. Schmitt, O. Kausiala, R. Tillmann, D. Zhao, C. Percival, T. J. Bannan, M. Priestley, Å. Hallquist, E. Kleist, A. Kiendler-Scharr, M. Hallquist, T. Berndt, G. McFiggans, J. Wildt, T. Mentel, M. Ehn, Multi-generation OH oxidation as a source for highly oxygenated organic molecules from aromatics. *Atmospheric Chemistry and Physics* 20, 515–537 (2020).
9. Z. Dong, R. Tang, H. Liu, Q. Zhang, W. Zong, J. Cheng, X. Shi, The formation mechanism of highly oxygenated organic molecules produced by toluene in the urban atmosphere. *Atmospheric Environment* 295, 119555 (2023).
10. X. Cheng, Q. Chen, Y. Jie Li, Y. Zheng, K. Liao, G. Huang, Highly oxygenated organic molecules produced by the oxidation of benzene and toluene in a wide range of OH exposure and NO x conditions. *Atmospheric Chemistry and Physics* 21, 12005–12019 (2021).
11. S. Wang, R. Wu, T. Berndt, M. Ehn, L. Wang, Formation of highly oxidized radicals and multifunctional products from the atmospheric oxidation of alkylbenzenes. *Environmental science & technology* 51, 8442–8449 (2017).
12. M. Wang, D. Chen, M. Xiao, Q. Ye, D. Stolzenburg, V. Hofbauer, P. Ye, A. L. Vogel,

R. L. I. Mauldin, A. Amorim, A. Baccarini, B. Baumgartner, S. Brilke, L. Dada, A. Dias, J. Duplissy, H. Finkenzeller, O. Garmash, X.-C. He, C. R. Hoyle, C. Kim, A. Kvashnin, K. Lehtipalo, L. Fischer, U. Molteni, T. Petäjä, V. Pospisilova, L. L. J. Quéléver, M. Rissanen, M. Simon, C. Tauber, A. Tomé, A. C. Wagner, L. Weitz, R. Volkamer, P. M. Winkler, J. Kirkby, D. R. Worsnop, M. Kulmala, U. Baltensperger, J. Dommen, I. El-Haddad, N. M. Donahue, Photo-oxidation of aromatic hydrocarbons produces low-volatility organic compounds. *Environmental science & technology* 54, 7911–7921 (2020).

1. J. Liu, Z. Liu, Z. Ma, S. Yang, D. Yao, S. Zhao, B. Hu, G. Tang, J. Sun, M. Cheng, Z. Xu, Y. Wang, Detailed budget analysis of HONO in Beijing, China: Implication on atmosphere oxidation capacity in polluted megacity. *Atmospheric Environment* 244, 117957 (2021).
2. R. M. Harrison, M. Dall’Osto, D. C. S. Beddows, A. J. Thorpe, W. J. Bloss, J. D. Allan, H. Coe, J. R. Dorsey, M. Gallagher, C. Martin, J. Whitehead, P. I. Williams, R. L. Jones, J. M. Langridge, A. K. Benton, S. M. Ball, B. Langford, C. N. Hewitt, B. Davison, D. Martin, F. Petersson, S. J. Henshaw, I. R. White, D. E. Shallcross, J. F. Barlow, T. Dunbar,F. Davies, E. Nemitz, G. J. Phillips, C. Helfter, C. F. Di Marco, S. Smith, Atmospheric chemistry and physics in the atmosphere of a developed megacity (London): an overview of the REPARTEE experiment and its conclusions. *Atmospheric Chemistry and Physics* 12, 3065–3114 (2012).
3. M. E. Jenkin, S. M. Saunders, M. J. Pilling, The tropospheric degradation of volatile organic compounds: a protocol for mechanism development. *Atmospheric Environment* 31, 81-104 (1997).
4. S. M. Saunders, M. E. Jenkin, R. G. Derwent, M. J. Pilling, Protocol for the development of the Master Chemical Mechanism, MCM v3 (Part A): tropospheric degradation of non-aromatic volatile organic compounds. *Atmospheric Chemistry and Physics* 3, 161-180 (2003).
5. M. E. Jenkin, K. P. Wyche, C. J. Evans, T. Carr, P. S. Monks, M. R. Alfarra, M. H. Barley, G. B. McFiggans, J. C. Young, A. R. Rickard, Development and chamber evaluation of the MCM v3. 2 degradation scheme for β-caryophyllene. *Atmospheric Chemistry and Physics* 12, 5275-5308 (2012).
6. D. B. Ingham, Diffusion of aerosols from a stream flowing through a cylindrical tube. *Journal of Aerosol Science* 6, 125-132 (1975).
7. Pichelstorfer, L. and Hofmann, W., Simulation of cigarette smoke dynamics in denuder tubes considering particle phase chemistry. *Aerosol Science and Technology* 51, 1419-1428 (2017).
